# 3-Mercaptopropanoic Acid-Doped Chitosan/Hybrid-Based Multilayer Sol-Gel Coatings for Cu Protection in 3.5% NaCl Solution

**DOI:** 10.3390/polym13213743

**Published:** 2021-10-29

**Authors:** Jaganathan Balaji, Tae Hwan Oh

**Affiliations:** School of Chemical Engineering, Yeungnam University, Gyeongsan 38541, Korea

**Keywords:** copper, chitosan, hybrid, 3-mercaptopropanoicacid, 3.5% NaCl

## Abstract

In this work, biopolymer based sol-gel was synthesized by doping 3-mercaptopropanoic acid (MPA) with chitosan and a hybrid of 3-glycidoxypropyltrimethoxysilane (GPTMS) and tetraethoxysilane (TEOS). Prepared MPA/hybrid-doped chitosan was applied toa copper (Cu) metal surface by the self-assembly technique to protect the Cu metal from corrosion in a 3.5% NaCl solution. The structure, mechanism and morphology of the modified electrodes were examined using Fourier transform infra-red (FT-IR) spectroscopy, X-ray photoelectron spectroscopy (XPS), scanning electron microscopy (SEM) with energy-dispersive X-ray spectroscopy (EDX), and atomic force microscopy (AFM). The decrease in surface roughness for Hy/chitosan/MPA-coated Cu indicates the formation of a dense layer on Cu metal confirmed by AFM. The corrosion protection evaluation of sol-gel coated electrodes was analyzed using electrochemical impedance spectroscopy (EIS) and potentiodynamic polarization studies (PDS) in a 3.5% NaCl medium. The MPA/hybrid-doped chitosan sol-gel coated Cu metal showed the greatest resistance to corrosionthanother sol-gel modified electrodes. The MPA-doped-chitosan/Hy sol-gel coating protected the Cu metal by an anodic dissolution process and improved its corrosion protection to 99.9%.

## 1. Introduction

Copper (Cu) and its alloys exhibit virtuous electrical, mechanical, and antifouling properties, corrosion resistance, excellent thermal, and electrical conductivity and play a vital role in thermal coolant systems and electronics industries [1,2,3]. However, corrosion of Cu in seawater and chloride causes material and economic losses around the world [4,5,6]. Therefore, it is important that copper is protected from corrosion in aggressive chloride environments. As a result, the demand for much greener, non-toxic coatings for the protection of Cu metal is increasing [7].

Physical vapor deposition (PVD), chemical vapor deposition (CVD), plasma spraying, electrodeposition, and sol-gel coatings are the various coating strategies applied to the corrosion protection of the Cu metal [8]. Compared to other methods, sol-gel based coatings have a high ability to protect against corrosion throughincreased corrosion resistance due to theirenvironmentalfriendliness, their excellent stability, and oxidation control [9,10]. Hybrid sol-gel coatings are the potential alternative to toxic current coatings like chromate and phosphate. Even though hybrid sol-gel coatings offer relatively dense films, some corrosive ions can still penetrate through the pores and corrode the copper metal. Thus, to overcome this disadvantage, the active biopolymer based on corrosion inhibitors can be incorporated into the sol-gel matrix for increased corrosion protection [11,12,13].

Chitosan, a biopolymer, has attracted many researchers and scientists due to its low cost, low toxicity, and environment-friendly nature, and has been used in a variety of medicinal applications [14]. Balaji et al. have studied hybrid zirconium and nano-TiO_2_ doped coating with water soluble chitosan biopolymer as a corrosion inhibitor to protect Al metals [15,16]. Several studies have reported on corrosion inhibitors as chitosan biopolymers for the protection of metal surfaces [17,18].

A self-assembled monolayer (SAM) is the formation of a dense film on Cu metal which acts as an effective barrier for corrosion protection [2]. Heterocyclic molecules of electron-rich compounds containing oxygen, nitrogen and sulphurcan reinforces SAM on metallic surfaces. Cu metal is more attractive to mercapto (-SH) functionalized compounds and forms a barrier layer that protects the metallic surface frompenetratingcorrosive ions [19].

In this research, we performed laboratory-scale experiments that showed effective protection oftheCu metal in the 3.5%NaCl. The results showed an inhibition efficiency of 99.9% by using a sol-gel coating of 3-mercaptopropanoic acid (MPA), 3-glycidoxypropyltrimethoxysilane (GPTMS), and tetraethoxysilane (TEOS) hybrid chitosan biopolymer.The results show promisethat this may be extended for the Cu metal used in the environment.

## 2. Materials and Methods

### 2.1. Chemicals

GPTMS with purity of 98%, TEOS with purity of 98%, chitosan, MPA with purity of 99%, sodium chloride (NaCl) with purity of 99%, and ethanol with purity of 99.5% were purchased from Sigma-Aldrich, Gangnam-Gu, Korea and used as received.

### 2.2. Cu Metal Polishing and Synthesis of Sol-Gel and Self-Assembly Coating

Cylindrical-shaped Cu metal electrode (a diameter of 1 cm) was polished using different grades of emery paper (1# to #7) and sonicated with acetone and ethanol for 30 min, and dried in a hot air oven.A hybrid (Hy) sol-gel matrix was prepared using ethanolic solutions of GPTMS and TEOS (2:1 molar ratio). Thiswas stirred in a reaction vessel for 6 h and its pH was adjusted to 4.0 by adding HCl to promote hydrolysis and condensation reaction. Hy/chitosan was prepared by adding a chitosan solution (5 wt%) to hybrid sol in the molar ratio of 1:1 and continuously stirred for a further 6 h at 40 °C for a ring opening reaction. Then Hy/chitosan/MPA was prepared by adding MPA to Hy/chitosan sol (1:1 molar ratio) and continuously stirred for further 6 h at 40 °C for crosslinking. The polished Cu metal immersed in prepared Hy/chitosan/MPA sol-gel for 12 h. For comparative study polished Cu metal also immersed in Hy and Hy/chitosan sol-gel for 12 h. Then, the sol-gel coated Cu electrodes were dried in airflow at 80 °C for 2 h.

### 2.3. Characterization of Modified/Unmodified Cu Electrodes

The chemical composition of bare Cu, Hy coated, Hy/chitosan coated and Hy/chitosan/MPA coated Cu electrodes were examined using Fourier transform-infrared (FT-IR) spectra (Perkin Elmer Spectrum at Core Research Support Center for Natural Products and Medical Materials in Yeungnam University) and X-ray photoelectron spectroscopy was carried out using a K-Alpha spectrometer (Thermo Scientific, Waltham, MA, USA). The surface morphological analysis of modified and unmodified Cu electrodes were studied using field emission scanning electron microscopy (FESEM, HitachiS-4800) and atomic force microscopy (AFM, Park system (XE 100) model).

The corrosion resistant efficiency of bare Cu, Hy-coated, Hy/chitosan-coated and Hy/chitosan/MPA-coated Cu electrodes were carried out using a CorrTest electrochemical workstation with conventional three-electrode set up. The electrochemical impedance spectroscopy (EIS) measurements were taken at the open circuit potential between the frequency ranges from 10 kHz to 0.01 Hz and the fitting results were obtained from ZSimpWin 3.21 software. The potentiodynamic polarization studies (PDS) were examined from −0.70 V to 0.2 V vs. Ag/AgCl at the scan rate of 1 mV s^−1^.

## 3. Results and Discussion

### 3.1. Fourier Transform Infrared (FT-IR) Analysis

**Figure 1:**
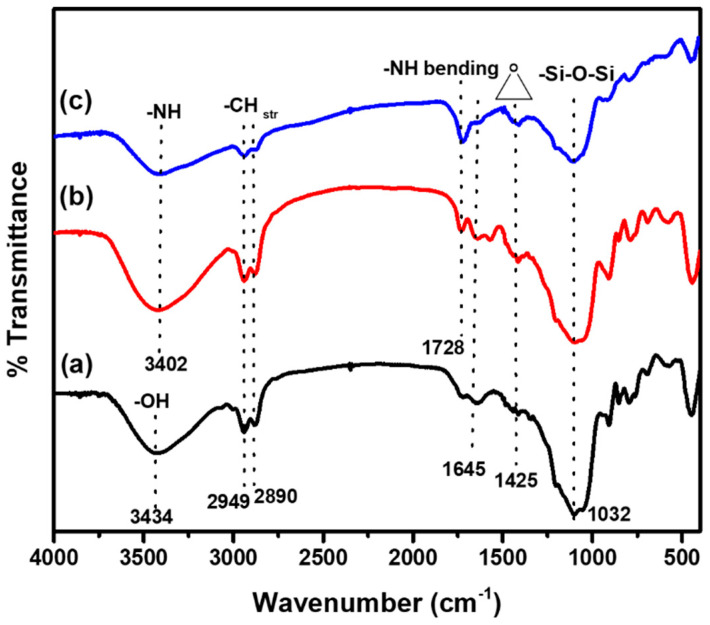
Fourier transform infrared (FT-IR) spectra of (**a**) Hy-coated, (**b**) Hy/chitosan-coated, and (**c**) Hy/chitosan/3-mercaptopropanoic acid (MPA)-coated Cu electrodes.

The FT-IR spectra of Hy coated, Hy/chitosan coated, and Hy/chitosan/MPA coated Cu electrodes are shown in Figure 1. In the FT-IR spectra of the Hy coated electrode, the band at 3434 cm^−1^, 2949 cm^−1^, 2890 cm^−1^, 1728 cm^−1^, 1645 cm^−1^, 1425 cm^−1^ and 1032 cm^−1^ are due to –OH, –CH stretching, C–C, epoxy group and Si–O–Si bond formation [20,21,22,23]. Additionally, for the Hy/chitosan- and Hy/chitosan/MPA-coated electrodes, the additional bands at 3402 cm^−1^ and 1728 cm^−1^ are due to –NH stretching and –NH bending. The absence of –SH stretching at 2632 cm^−1^ confirms the doping of chitosan and MPA in the hybrid sol-gel matrix [24,25].

### 3.2. X-ray Photoelectron Spectroscopy (XPS) Analysis

**Figure 2:**
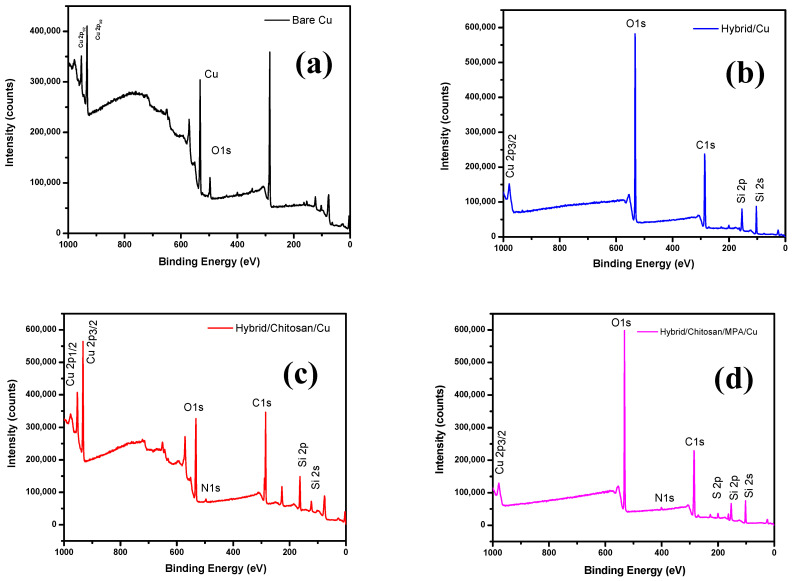
X-ray photoelectron spectroscopy (XPS) spectra of (**a**) bare Cu, (**b**) Hy-coated, (**c**) Hy/chitosan-coated, and (**d**) Hy/chitosan/MPA-coated Cu electrodes.

**Figure 3:**
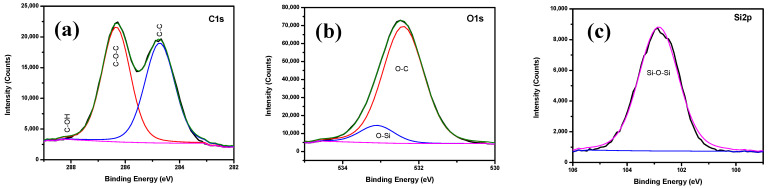
Deconvoluted XPS spectra of Hy-coated Cu electrodes; (**a**) C 1s, (**b**) O 1s, and (**c**) Si 2p.

The XPS spectra shown in Figure 2 were used to determine the chemical composition of bare Cu, Hy coated, Hy/chitosan coated, and Hy/chitosan/MPA coated Cu electrodes. The peaks of bare Cu shows three peaks at 497.7, 933.6, and 952.5 eV due to the presence of O 1s, Cu 2p_3/2_, and Cu 2p_1/2_, respectively. For the Hy-coated electrode, the peaks are shown at 104.6, 153.6, 286.1, 534.1, and 980.1 eV due to the presence of Si 2s, Si 2p, C 1s, O 1s, and Cu 2p_3/2_, which confirm the presence of the hybrid sol-gel coating on copper surface. The peaks of Hy/chitosan coated electrode are shown at 122.2, 162.5, 283.4, 496.3, 534.1, 933.6, and 980.1 eV due to the presence of Si 2s, Si 2p, C 1s, N 1s, O 1s, Cu 2p_3/2_, and Cu 2p_1/2_, confirming the doping of chitosan in the hybrid sol-gel matrix. Furthermore, the Hy/chitosan/MPA-coated electrode shows peaks at 99.6, 153.6, 162.5, 284.7, 400.5, 531.6, and 977.6 eV due to the presence of Si 2s, Si 2p, S 2p, C 1s, N 1s, O 1s, and Cu 2p_3/2_. The expanded XPS spectrum of the Hy-coated electrode (Figure 3) shows C 1s peaks consistingof three binding peaks at 284.7, 286.3, and 288.2 eV due to the presence of C–C, C–O–C, and C–OH functional groups. The XPS of O 1s level shows two binding peaks at 532.4 eV and 533.1 eV due to the presence of C–O–C and Si–O functional groups. The XPS spectrum of Si 2p shows one binding peaks at 102.8 eV due to the presence of Si–O–Si bond formation confirming the hydrolysis and condensation reaction of coated hybrid sol-gel on the Cu metal [26].

**Figure 4:**
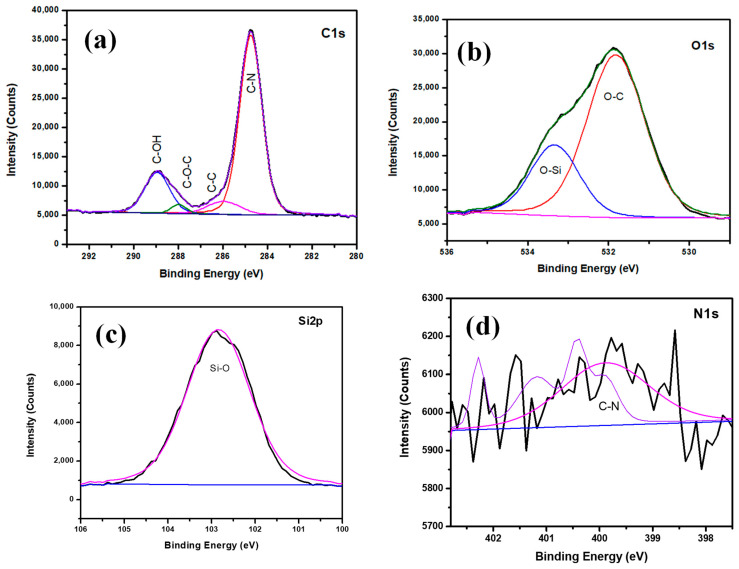
Deconvoluted XPS spectra of Hy/chitosan coated electrodes; (**a**) C 1s, (**b**) O 1s, (**c**) Si 2p, and (**d**) N 1s.

The expanded XPS spectrum of Hy/chitosan-coated (Figure 4) shows C 1s peaks consisting of four binding peaks at 284.7, 286.1, 287.9, and 288.9 eV due to the presence of C–N, C–C, C–O–C, and C–OH functional groups. The O 1s level shows two binding peaks at 531.8 and 533.3 eV due to the presence of C–O–C and Si–O groups. The Si 2p shows one binding peak at 102.8 eV due to the presence of Si–O–Si bond formation. The N 1s shows one binding peaks at 399.9 eV due to the presence of C–N bond formation confirming the doping of chitosan into the hybrid sol-gel matrix and the coating on Cu metal.

**Figure 5:**
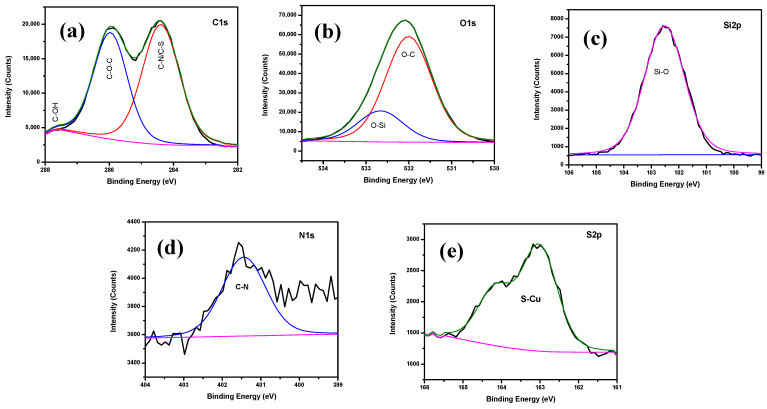
Deconvoluted XPS spectra of Hy/chitosan/MPA coated electrodes; (**a**) C 1s (**b**) O 1s (**c**) Si 2p (**d**) N 1s and (**e**) S 2p.

In the expanded XPS spectrum of Hy/chitosan/MPA-coated electrode (Figure 5), similar peaks to those of Hy/chitosan-coated electrodes are shown and additionally, the peak of S 2p is shown for one binding peaks at 163.1 eV due to the presence of Cu–S (thiolate bond) formation, confirming the doping of MPA into Hy/chitosan sol-gel matrix and the coating on Cu metal.

The calculated atomic weights (%)for Hy/chitosan/MPA sol-gel are C (47.6%), O (37.97%), Si (11.64%), N (1.37%), S (1.33%), and Cu (0.09%) [10]. The XPS analysis confirmed the formation of an MPA/chitosan/Hybrid sol-gel layer on the Cu metal.

### 3.3. Scanning Electron Microscopy (SEM) and Energy-Dispersive X-ray Spectroscopy (EDX) Analysis

**Figure 6:**
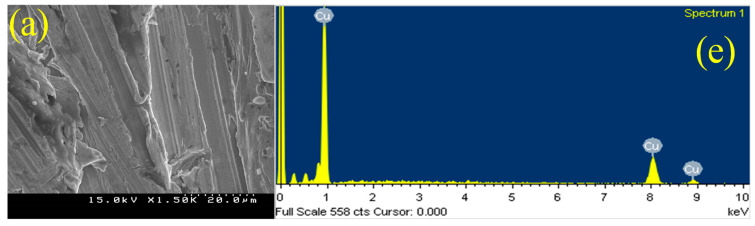

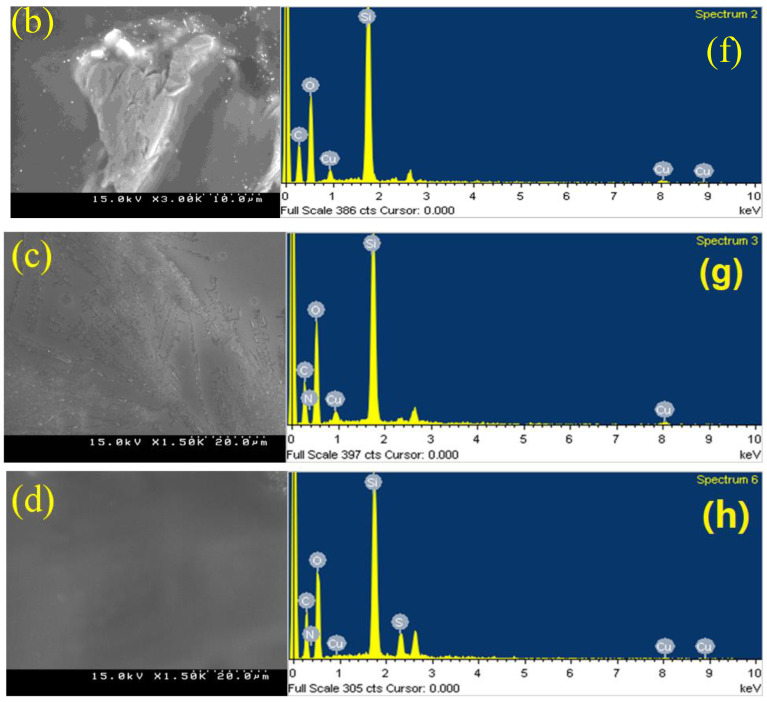
Scanning electron microscopy (SEM) images and energy-dispersive X-ray spectroscopy (EDX) of bare Cu (**a**,**e**), Hy coated (**b**,**f**), Hy/chitosan coated (**c**,**g**), and Hy/chitosan/MPA coated Cu electrodes (**d**,**h**).

The SEM photographs for the bare Cu, Hy coated, Hy/chitosan coated, and Hy/chitosan/MPA coated Cu electrodes are presented in Figure 6a–d, respectively. The SEM image of bare Cu (Figure 6a) shows a lot of scratches due to polishing the Cu surfaces, while Hy coated electrode (Figure 6b) shows a poor adhesive layer on the Cu metal due to unstable Cu–O–Si bond formation [10,21,25,27]. Hy/chitosan-coated (Figure 6c) electrode shows uniform distribution of chitosan layer.The hybrid sol-gel coated and Hy/chitosan/MPA-coated Cu electrodes show (Figure 6d) a dense and compactly packed adhesive layer that can protect the copper metal from corrosion.

The EDAX results for the bare Cu, Hy-coated, Hy/chitosan-coated, and Hy/chitosan/MPA-coated Cu electrodes are shown in Figure 6e–h, respectively. In bare Cu (Figure 6e) the peaks at 1, 8 and 9 keV for Cu metal, while the Hy-coated (Figure 6f) electrode shows peaks at 0.25, 0.50, 1.0, and 1.8 keV due to presence of C, O, Cu and Si confirming the coating of hybrid sol-gel. In the Hy/chitosan-coated electrode (Figure 6f), the additional peak of N at 0.30 keV is shown, confirming the doping of chitosan into a hybrid sol-gel matrix. For the MPA with Hy/chitosan sol-gel, a new additional peak of S at 2.15 keV is shown, confirming the doping of MPA and the forming a protective layer on Cu metal.

### 3.4. Atomic Force Microscopy (AFM) Analysis

**Figure 7:**
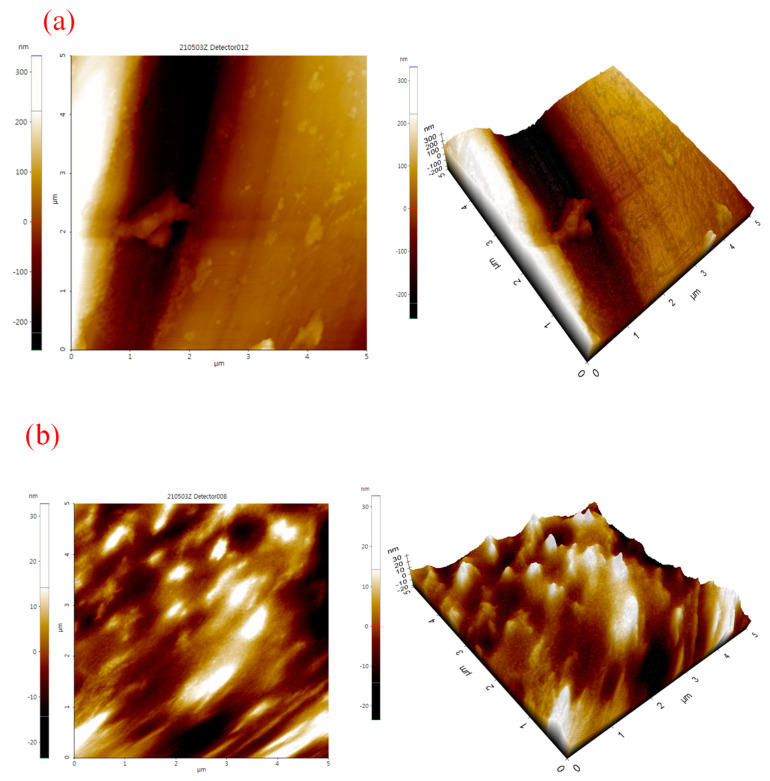
Atomic force microscopy (AFM) images of (**a**) bare Cu and (**b**) Hy/chitosan/MPA-coated Cu electrode.

The AFM images of bare Cu and Hy/chitosan/MPA-coated Cu electrodes are shown in Figure 7. The bare Cu shows scratches same as the SEM image. For the Hy/chitosan/MPA-coated Cu electrode (Figure 7b), a uniform adhering layer is shown. Average surface roughness (S_a_) and root mean square (S_q_) of the Hy/Chitosan/MPA-coated electrode are lower than those of bare Cu (Table 1). The decrease in surface roughness of Hy/Chitosan/MPA-coated Cu indicates the formation of a densely packed layer on the Cu metal [27].

### 3.5. Electrochemical Impedance Spectroscopy (EIS)

**Figure 8:**
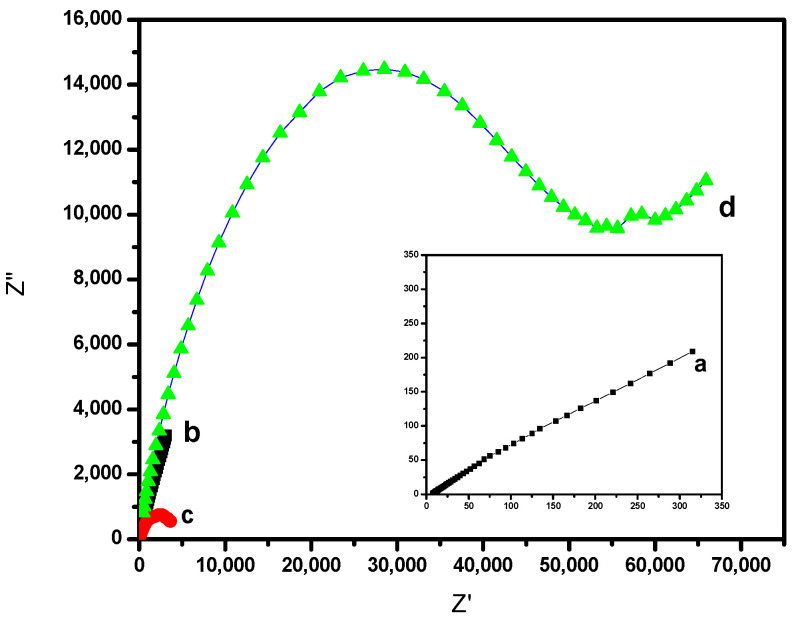
Electrochemical impedance spectroscopy (EIS) of (**a**) bare Cu (inset), (**b**) Hy-coated, (**c**) Hy/chitosan-coated, and (**d**) Hy/chitosan/MPA-coated Cu electrodes in 3.5% NaCl solution.

**Figure 9:**
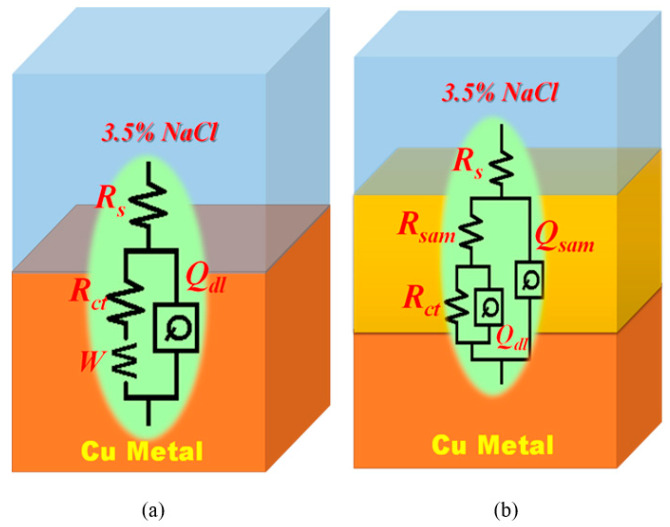
Equivalent circuits of (**a**) bare Cu and (**b**) coated Cu substrates.

EIS for Cu metal, Hy-coated, Hy/chitosan-coated, and Hy/chitosan/MPA-coated Cu electrodes was analyzed after immersing them in 3.5% NaCl solution for 1 h and the corresponding Nyquist plot is shown in Figure 8. The results were fitted using an equivalent circuit shown in Figure 9 and the fitted parameters are shown in Table 2. In the equivalent circuit for bare Cu (Figure 9a), *R_s_* is the solution resistance, *R_ct_* the charge transfer resistance, *W* the Warburg impedance, and *Q_dl_* the double layer capacitance, respectively. For sol-gel modified Cu (Figure 9b), *R_sam_* and *Q_sam_* are coated resistance and coated double layer capacitance, respectively [10].

The corrosion protection efficiency of bare Cu and modified Cu electrodes in the EIS analysis were calculated using Equation (1) [28].

(1) $$IE\left(EIS\right)\left(\%\right)=\frac{R_{ct}^{s}-R_{ct}^{b}}{R_{ct}^{b}}\times 100$$

where, $R_{ct}^{s}$ and $R_{ct}^{b}$ are the are the charge transfer resistance of the modified and unmodified Cu electrodes, respectively.

The Hy/chitosan/MPA-coated Cu electrode shows the largest $R_{ct}^{s}$ values compared to bare Cu, Hy-coated, and Hy/chitosan-coated layers, and hence the Hy/chitosan/MPA-coated Cu electrode showed the highest corrosion inhibition efficiency of 99.9%. The Hy/chitosan/MPA acts as a very good protective layer against the diffusion of corrosive chloride ions into Cu metal surface.

### 3.6. Potentiodynamic Polarization Studies (PDS)

**Figure 10:**
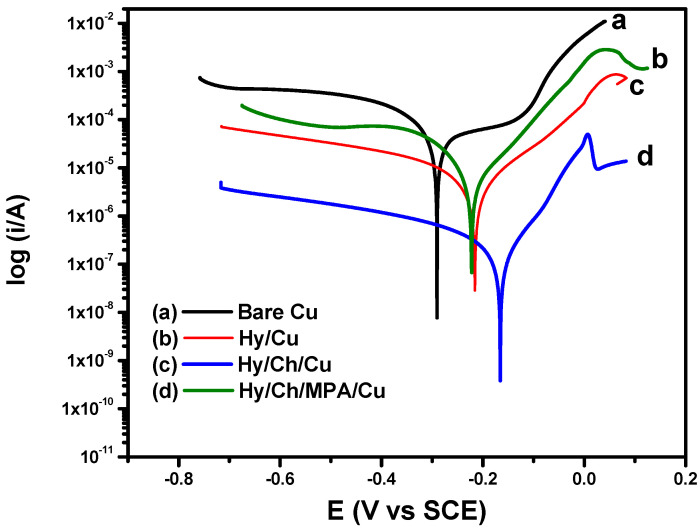
Potentiodynamic study(PDS) of (**a**) bare Cu, (**b**) Hy-coated, (**c**) Hy/chitosan-coated, and (**d**) Hy/chitosan/MPA-coated Cu electrodes in 3.5% NaCl solution.

From PDS analysis, the corresponding Tafel plot is shown in Figure 10 and the Tafel parameters are summarized in Table 3. The corrosion inhibition efficiency for unmodified and modified Cu electrodes in the Tafel plot were calculated using Equation (2) [29,30].

(2) $$IE\left(PDS\right)\left(\%\right)=\frac{i_{corr}^{b}-i_{corr}^{s}}{i_{corr}^{b}}\times 100$$

where, $i_{corr}^{b}$ and $i_{corr}^{s}$ are the corrosion current of the unmodified and modified Cu metals, respectively.

The further decrease in $i_{corr}^{s}$ value for Hy/chitosan/MPA-coated Cu electrodes was observed due to doping of MPA and chitosan with extended silane linkages. Hy/chitosan/MPA-coated Cu electrodes shows extended anodic shifts compared to Hy/Cu- and Hy/chitosan sol-gel coated electrodes. Hy/chitosan/MPA coating protects the Cu electrodes against anodic oxidation reaction and the formation of an adhesive barrier layer against the attack of aggressive chloride ions [21,27].

### 3.7. Mechanism

**Figure 11:**
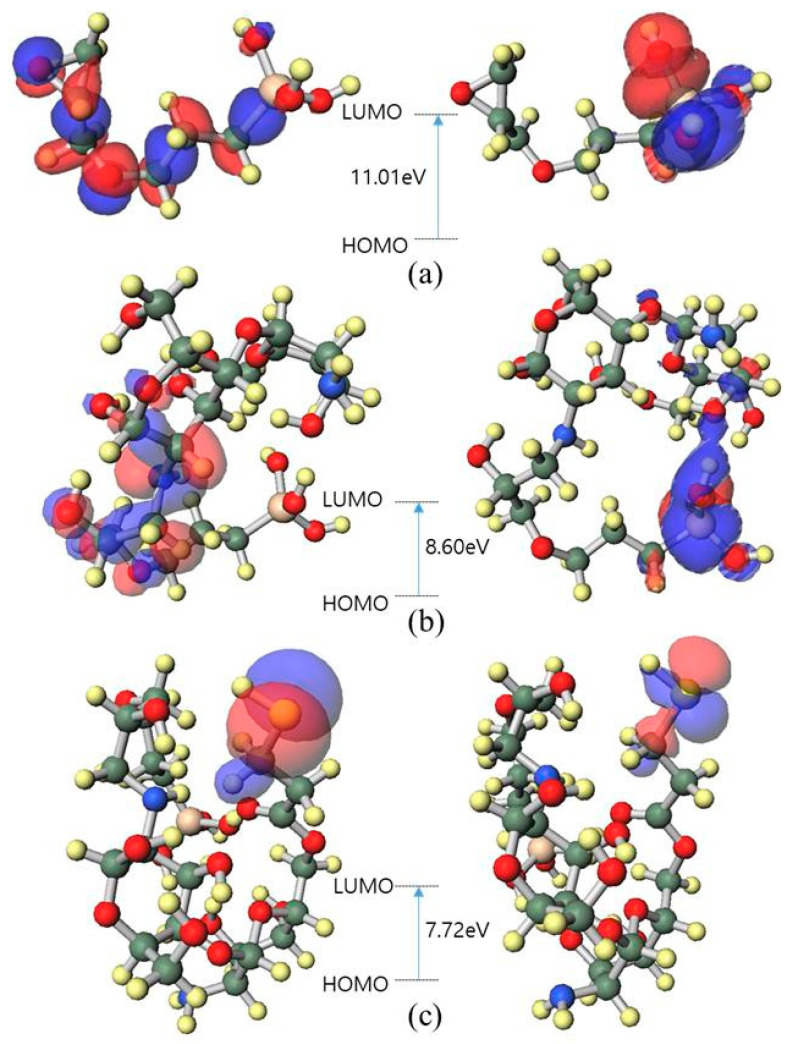
Highest occupied molecular orbital (HOMO) and lowest unoccupied molecular orbital (LUMO) band gap energy of (**a**) Hy-coated, (**b**) Hy/chitosan-coated, and (**c**) Hy/chitosan/MPA-coated, respectively.

Density functional theory (DFT)was computed using a semi-empirical molecular orbital package (MOPAC) with PM7 Hamiltonian. The highest occupied molecular orbital (HOMO) energy and the lowest unoccupied molecular orbital (LUMO) energy were calculated and are presented in Figure 11. The energy of the Hy/chitosan/MPA is the smallest value of 7.72 eV. The more stable inhibitor with a lower HOMO-LUMO gap energy leads to a higher corrosion efficiency [31]. A higher HOMO energy level reduces the HOMO-LUMO gap energy and induces organic molecules to give free electrons to the metal surface, resulting in a high inhibition efficiency.

**Figure 12:**
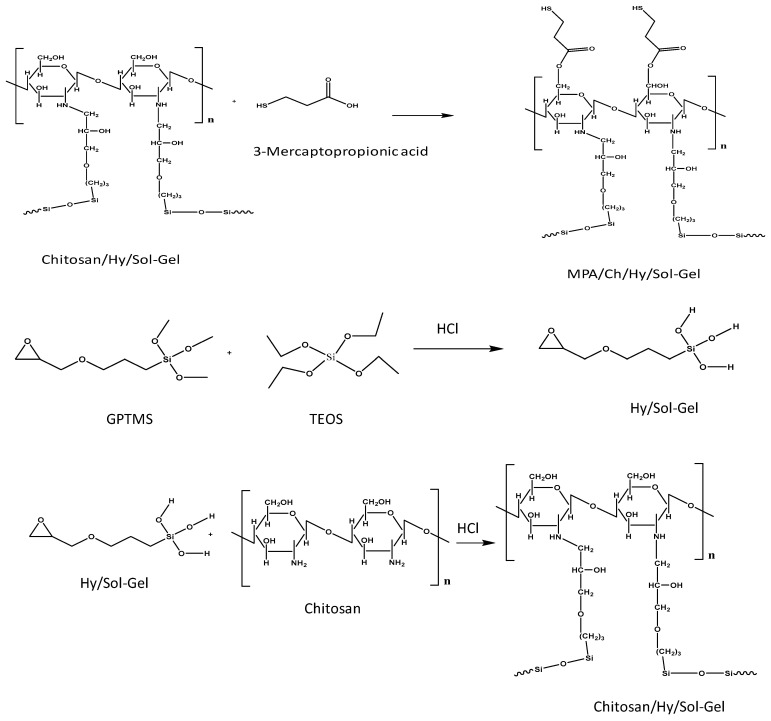

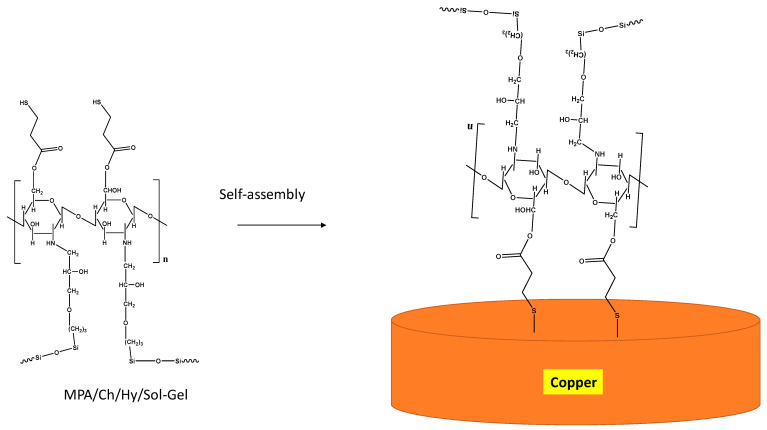
Proposed mechanism of Hy/chitosan/MPA sol-gel coating on Cu metal.

The schematic representation of Hy/chitosan/MPA sol-gel coated layer on Cu metal is shown in Figure 12. The acidic carbon in the hybrid sol-gel undergoes nucleophilic attack by the addition of chitosan biopolymer leading to the opening of the epoxy ring and the formation of the Si–O–Si linkage. The addition of 3-mercaptopropanoicacid (MPA) to chitosan/hybrid sol-gel induces an addition reaction by the elimination of water. This sol-gel matrix was grafted onto Cu metal by a self-assembly method leading to the formation of a Cu–S (thiolate) bond. Thus, the MPA-doped chitosan/Hy sol-gel layer protects the Cu metal from corrosion in a 3.5% NaCl solution [32,33].

## 4. Conclusions

Chitosan biopolymer based sol-gel doping with 3-mercaptopropanoicacid (MPA) and hybrids is a successful strategy to protect Cu metal surface. Chemical composition analyses like FT-IR and XPS revealed the formation of Si–O–Si, Cu–S, and epoxy ring opening, confirming the doping of chitosan biopolymer and MPA to the hybrid sol-gel matrix. SEM and AFM analyses confirmed the formation of an adhesive homogenous layer of Hy/chitosan/MPA sol-gel coated layer on Cu metal. The EIS and PDS results showed that the corrosion inhibition efficiency of an MPA-doped chitosan/Hy sol-gel coated layer is 99.9%. A very high corrosion inhibition of the MPA-doped chitosan/Hy sol-gel coated layer was achieved, and it can be used in industrial application to protect Cu metal in a NaCl solution.

## Figures and Tables

**Table 1:** AFM roughness parameters (nm) for (a) bare Cu and (b) Hy/chitosan/MPA-coated Cu electrodes.

Nature of Sol-Gel Coating	Average Surface Roughness (Sa) (nm)	Root Mean Square (Sq) (nm)
Bare Cu	123.4	146.0
Hy/Chitosan/MPA/Cu	4.6	6.2

**Table 2:** Electrochemical impedance parameters for bare Cu, Hy-coated, Hy/chitosan-coated, and Hy/chitosan/MPA-coated Cu electrodes in 3.5% NaCl solution.

Nature of Sol-Gel Coatings	*−E_corr_* (mV)	*i_corr_* (µA·cm^−2^)	η_PDS_ (%)
Bare Cu	−292	7.09 × 10^−4^	-
Hy/Cu	−222	1.18 × 10^−4^	83.3
Hy/Chitosan/Cu	−215	7.05 × 10^−5^	90.0
Hy/Chitosan/MPA/Cu	−165	4.08 × 10^−7^	99.9

**Table 3:** Tafel parameters for (a) bare Cu, (b) Hy-coated, (c) Hy/chitosan-coated, and (d) Hy/chitosan/MPA-coated Cu electrodes in 3.5% NaCl solution.

Parameters	Bare Cu	Hy/Cu	Hy/Chitosan/Cu	Hy/Chitosan/MPA/Cu
*R_s_* (Ω)	5.0	6.2	9.0	10.0
*Q_sam_* (µF cm^−2^)	-	2.25 × 10^−5^	1.01 × 10^−5^	3.11 × 10^−7^
*n*_1_	-	0.70	0.80	0.73
*R_sam_* (Ω cm^2^)	-	30.5	48.3	76.2
*Q_dl_* (µF cm^−2^)	1.17 × 10^−3^	1.68 × 10^−5^	1.53 × 10^−5^	1.55 × 10^−5^
*n*_2_	0.80	0.80	0.53	0.85
*R_t_* (kΩ)	586.4	3557	6256	9,764,000
*η* (%) using *R_t_*	-	83.5	90.6	99.9
